# P045 Occult large vessel vasculitis in a patient with apparent rheumatoid arthritis

**DOI:** 10.1093/rap/rkag113.075

**Published:** 2026-11-03

**Authors:** Hugh Larkin, Suzanne Lane, Haris Raouf

**Affiliations:** East Suffolk and North Essex NHS Foundation Trust, Ipswich, United Kingdom; East Suffolk and North Essex NHS Foundation Trust, Ipswich, United Kingdom; University of Cambridge, Cambridge, United Kingdom

## Abstract

**Introduction:**

We present the case of a 66-year-old female whose clinical picture evolved over two decades. She initially presented with asymmetric peripheral inflammatory arthritis and was treated as seronegative arthritis. Ten years later, she developed markedly elevated rheumatoid factor and anti-CCP antibodies and was treated as seropositive rheumatoid arthritis. She subsequently reported significant weight loss with persistently raised inflammatory markers but no synovitis. Endoscopy and CT excluded malignancy. PET-CT demonstrated increased uptake in the right carotid and left internal iliac arteries, consistent with large vessel vasculitis. She responded well to corticosteroid and tocilizumab.

**Case description:**

This patient first presented in 2010 with inflammatory pain affecting the MCP, PIP and DIP joints, associated with minimal swelling and morning stiffness. Investigations at that time revealed a weakly positive ANA, with normal inflammatory markers, negative anti-CCP antibodies and imaging consistent with osteoarthritis. She was initially diagnosed with inflammatory osteoarthritis and responded to hydroxychloroquine.

Over subsequent years, persistent inflammatory symptoms led to escalation of therapy with methotrexate, followed by sulfasalazine for evolving seronegative inflammatory arthritis. This was thought to represent psoriatic arthritis. Etanercept was commenced in 2017, producing an excellent clinical response for several years.

Between 2023 and 2025, her disease course changed significantly. She experienced recurrent episodes of acute inflammatory arthritis labelled as gout, with serum urate 488 µmol/L and CRP >100 mg/L. Repeat serology became strongly positive for rheumatoid factor (403 IU/mL) and anti-CCP antibodies (317 U/mL), consistent with seropositive rheumatoid arthritis. During the same period, she developed microcytic anaemia, pancytopenia, declining renal function and unintentional weight loss. Extensive investigations, including CT imaging, colorectal, respiratory, haematological and ENT assessment, eventually excluded malignancy.

She subsequently developed polymyalgia rheumatica-like symptoms and persistently elevated inflammatory markers. PET-CT demonstrated large vessel vasculitis involving the carotid arteries, thoracic aorta and common iliac arteries. Etanercept was switched to tocilizumab to treat both rheumatoid arthritis and large-vessel vasculitis.

**Discussion:**

This was a sequential diagnostic challenge as the patient’s presentation and serology evolved over fifteen years. Initially, an undifferentiated seronegative inflammatory arthritis was considered to represent inflammatory osteoarthritis, but later psoriatic arthritis. The changing RF and anti-CCP antibody status then led to a diagnosis of seropositive rheumatoid arthritis. However, as constitutional symptoms began to dominate over improving arthralgia, it was important to consider other aetiologies beyond active inflammatory arthritis.

Our case highlights that patients’ diagnoses can indeed change with time and that it is important to remain vigilant as symptoms evolve.

Rheumatoid vasculitis tends to favour the medium and small vessels rather than involving the aorta and its major branches. Large vessel vasculitis in patients with rheumatoid arthritis is described but is not frequently encountered. In cases such as this, where a patient with pre-existing inflammatory arthritis develops prominent systemic symptoms, vasculitis should be considered as a potential cause.

The patient’s outcome also demonstrates how tocilizumab can be used to treat large-vessel vasculitis and concurrent rheumatoid arthritis effectively.

**Key learning points:**

1. Inflammatory arthritis may change, and the diagnosis should be reconsidered as new clinical features or investigation abnormalities emerge.

2. Seroconversion to rheumatoid factor and anti-CCP positivity can develop many years after the original symptom onset.

3. Constitutional symptoms in patients with inflammatory arthritis should act as a stimulus to investigate thoroughly for infection, malignancy and large vessel vasculitis, rather than simply being attributed to active disease.

4. Tocilizumab can be effective in treating large vessel vasculitis with concurrent rheumatoid arthritis.

